# Supplementary material for: pH-Responsive Super-Porous Hybrid Hydrogels for Gastroretentive Controlled-Release Drug Delivery
Source: Pharmaceutics. 2023 Mar 2;15(3):816. doi: 10.3390/pharmaceutics15030816 (PMC10053105; doi:10.3390/pharmaceutics15030816)
Supplement: Supplementary file 1 [file pharmaceutics-15-00816-s001.zip › pharmaceutics-2175653-supplementary.docx]

Supplementary Materials: pH-responsive Super-Porous Hybrid Hydrogels for Gastroretentive Controlled-release Drug Delivery

**Ajkia Zaman Juthi, Fenfen Li, Bo Wang, Md Mofasserul Alam , Md Eman Talukder , and Bensheng Qiu**

Stock solution preparations

Glacial acetic acid buffer (0.1 M, pH 5.0) preparation:

- Prepare 800 mL of deionized water in a suitable container.
- Add 5.772 g of sodium acetate to the solution.
- Add 1.778 g of glacial acetic acid to the solution.
- Adjust the solution to the preferred pH using 10 M HCl (typically pH ≈ 5.0).
- Add deionized water until the volume is 1 L.

Glacial acetic acid buffer (0.1 M, pH 5.0) preparation:

- Dissolve 20.0 g NaOH pellets in 80 ml deionized water in a beaker.
- When cooled, bring the final volume to 100 ml.
- Transfer the solution to a glass bottle and label the reagent.

Standard calibration curve

The absorbance values of amoxicillin trihydrate (AT) were measured at a λ-Max of about 272 nm (**Figure S7**). A total of 50 mg of the drug was accurately weighed and dissolved in a few ml of 0.1 N HCl in a 100 ml volumetric flask and further diluted to 100 ml with 0.1 N HCl to produce a 1 mg/ml stock solution. A stock solution was prepared from the standard solution to give a concentration of 100 µg/ml in 0.1 N HCl. Amounts of 0.5, 1, 1.5, 2, 2.5,3, and 3.5 ml were pipetted out for the 10 ml volumetric flasks. Hence, the volume was made up of the drug with 0.1 N HCl and produced 9, 14, 19, 24, 29, 34, and 40 µg/ml of standard dilutions for the experiment [1].

**Figure S1.** The mechanistic explanation for the probable synthesis of the SPHHs. Proposed crosslinking of pectin–2HEMA–N,N-MDAc–poloxamer-407; preparation of pectin (hybrid agent) and monomer hydroxyethyl methacrylate (2HEMA) followed by the synthesis of SPHHs from N,N-methylene-bis-acrylamide (BIS) (crosslinking agent), APS (ammonium persulfate), TEMED (N,N,N,N-tetramethylenediamine) (polymerization initiator pairs), pluronic F127/poloxamer 407 (foam stabilizers), and sodium bicarbonate (foaming agents) [2-4].


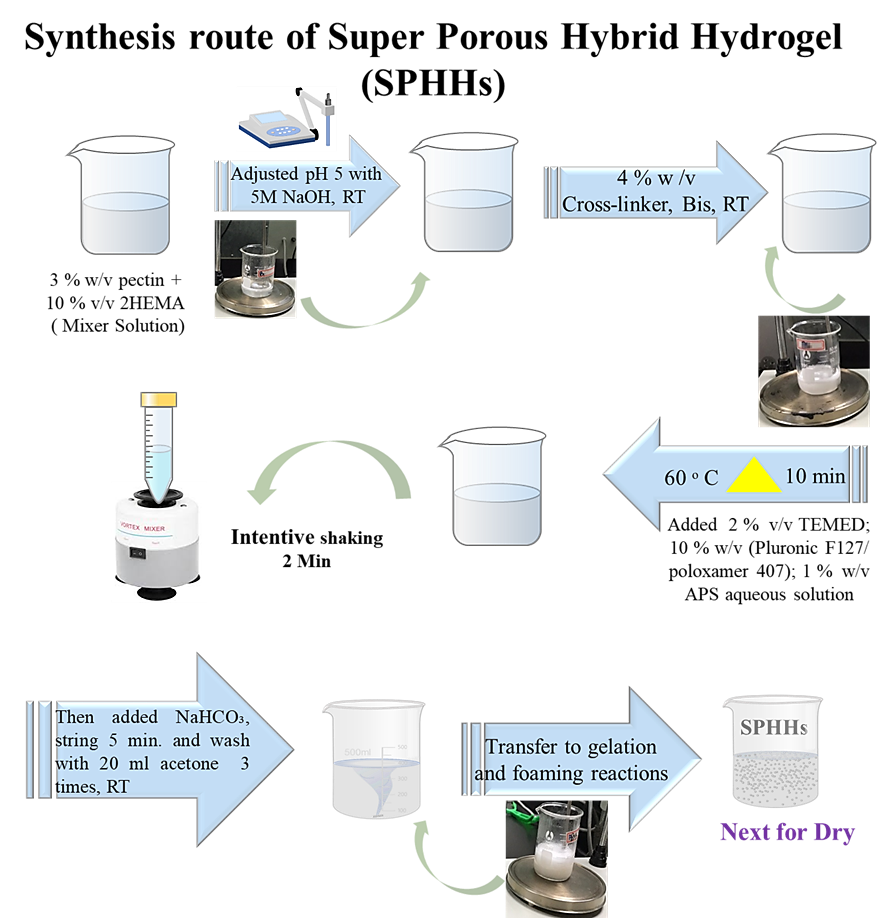


**Figure S2.** Schematic representation of the synthesis procedure of a semi-IPN for SPHHs [5].


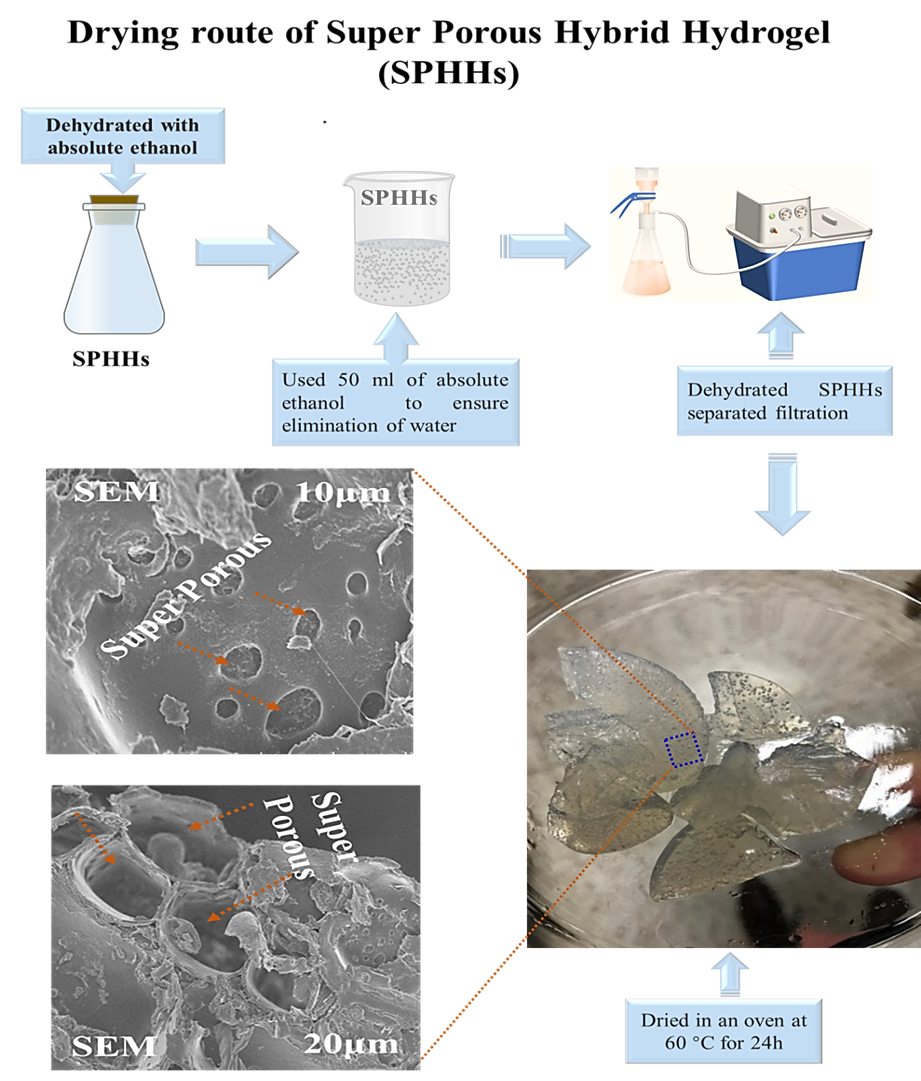


**Figure S3.** Schematic representation of the drying of super-porous hybrid hydrogels comprising dehydration, filtration, and drying procedures [6].


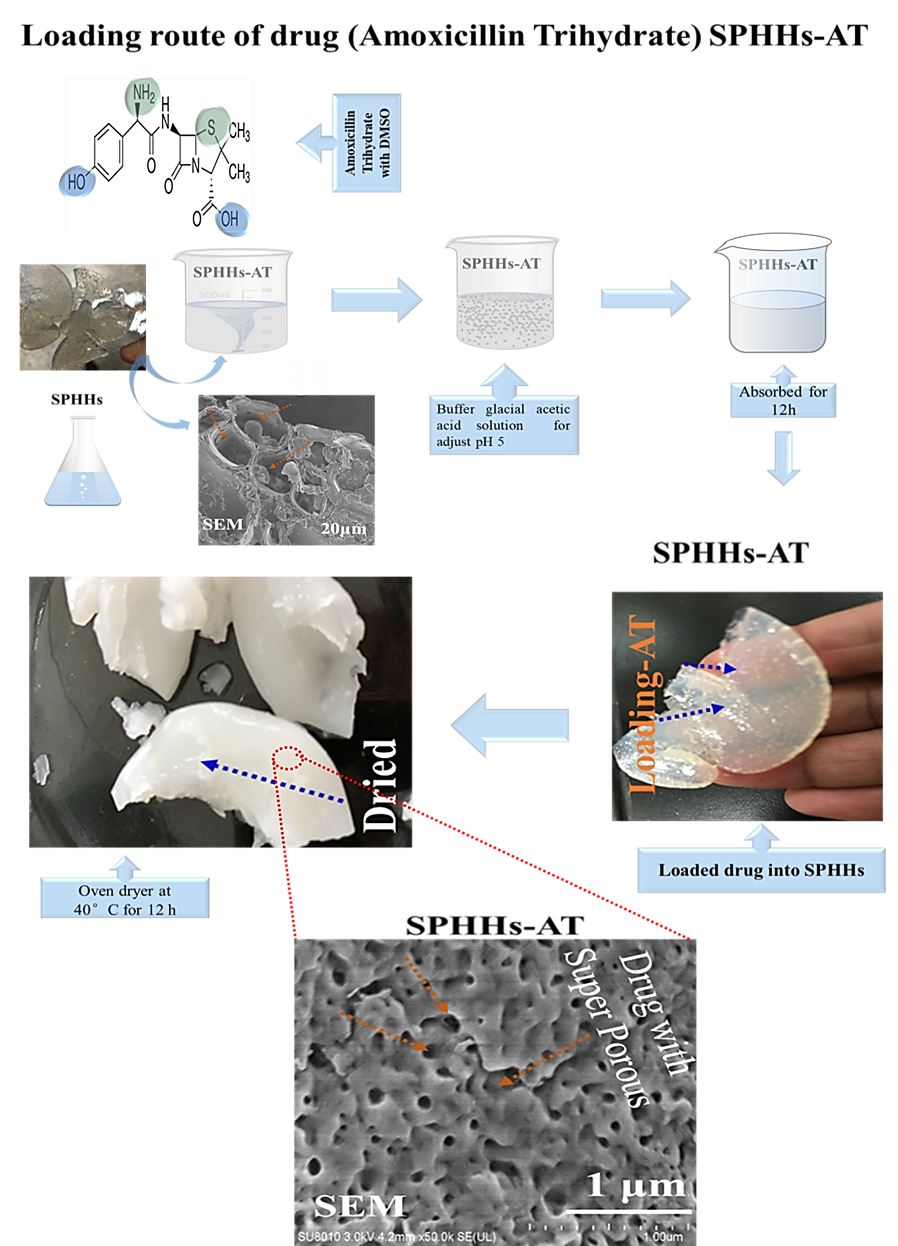


**Figure S4.** Schematic design of the drug-loading process of SPHHs-AT absorption and drying.


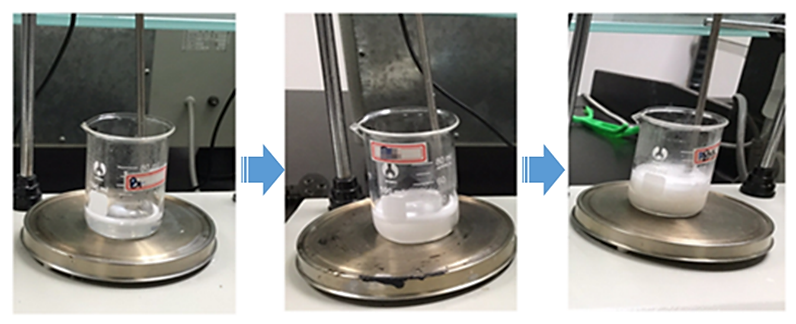


**Figure S5.** Super-porous hybrid hydrogels synthesis procedure steps.


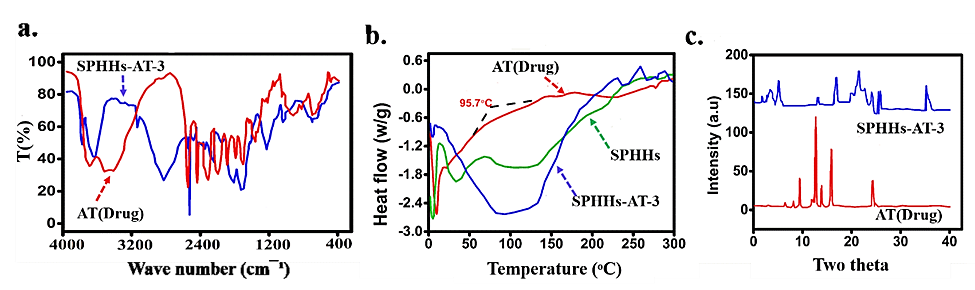


**Figure S6.** Overlay diagram of (**a**) FTIR spectra of SPHHs-AT-3 and AT; (**b**) DSC thermograms of AT, SPHHs, and SPHHs-AT-3; (**c**) X-ray diffraction of AT and SPHHs-AT-3.

**Figure S7.** Calibration curve; absorbance values of AT were measured at a λ_max_ of 272 nm [7].


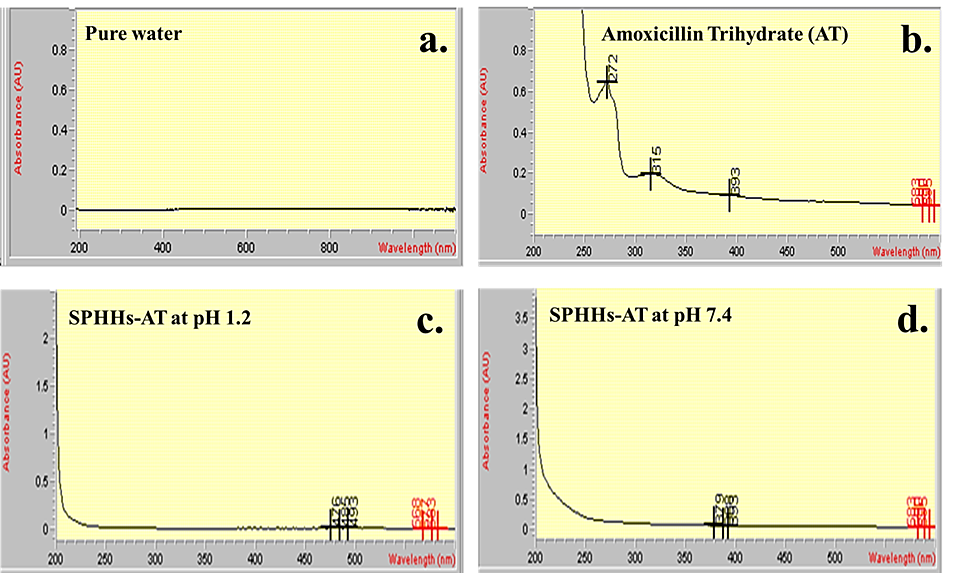


**Figure S8.** UV spectrum of (**a**) water ; (**b**) amoxicillin trihydrate (AT); (**c**) SPHHs-AT with pH 1.2 ; (**d**) SPHHs-AT with pH 7.4 [8-10].

**Table S1.** Synthesis of semi-IPN super-porous hybrid hydrogels (SPHHs)^†^ of amoxicillin trihydrate (AT).

| **Ingredient** | **SPHHs-AT-1** | **SPHHs-AT-2** | **SPHHs-AT-3** | **SPHHs-AT-4** | **SPHHs-AT-5** | **SPHHs-AT-6** | **SPHHs-AT-7** |
| --- | --- | --- | --- | --- | --- | --- | --- |
| Pectin ^a^ | 4 | 6 | 8 | 4 | 4 | 4 | 4 |
| 2HEMA^b^ | 4 | 4 | 4 | 6 | 8 | 4 | 4 |
| BIS ^c^ | 1 | 1 | 1 | 1.5 | 2 | 2.5 | 3 |
| F127 ^d^ | 5 | 5 | 5 | 5.5 | 5 | 4.5 | 4 |
| APS ^e^ | 0.1 | 0.1 | 0.1 | 0.1 | 0.1 | 0.1 | 0.1 |
| TEMED^f^ | 0.1 | 0.1 | 0.1 | 0.1 | 0.1 | 0.1 | 0.1 |
| NaCO3 ^mg^ | 80 | 80 | 80 | 80 | 80 | 80 | 80 |
| AT ^mg^ | 50 | 50 | 50 | 50 | 50 | 50 | 50 |

Herein,.
**^a^** 3% w/v in ml, **^b^** 10% v/v in ml, **^c^** 4% w/v in ml, **^d^** 10% w/v in ml pluronic F127/poloxamer 407, **^e^** 1% w/v in ml, **^f^** 2% v/v in ml.
**^†^** semi-IPN super-porous hybrid hydrogels synthesis [11,12].

**Table S2.** pH-dependent swelling appearance of SPHHs-AT.

| **SPHHs-AT** | Time ^min^ | pH-1.2 (AV) | pH-1.2 (SD) | Time ^min^ | pH-7.4 (AV) | pH-7.4 (SD) |
| --- | --- | --- | --- | --- | --- | --- |
| 3 | 0 | 0.4 | 0.2 | 30 | 88.66 | 1.25 |
| 2 | 5 | 23.66 | 1.52 | 35 | 70 | 2 |
| 1 | 10 | 36.5 | 1.5 | 40 | 54 | 2 |
| 4 | 15 | 64.33 | 1.52 | 45 | 35.5 | 1.80 |
| 5 | 20 | 72.66 | 1.52 | 50 | 21.16 | 1.25 |
| 6 | 25 | 80.23 | 0.87 | 55 | 10.16 | 1.25 |
| 7 | 30 | 83.66 | 1.60 | 60 | 8.66 | 1.52 |

**Table S2. (**Continued)

| **SPHHs-AT** | Time ^min^ | pH-1.2 (AV) | pH-1.2 (SD) | Time ^min^ | pH-7.4 (AV) | pH-7.4 (SD) |
| --- | --- | --- | --- | --- | --- | --- |
| 3 | 60 | 8.66 | 1.52 | 90 | 87.66 | 2.51 |
| 2 | 65 | 27.66 | 1.52 | 95 | 67.5 | 2.17 |
| 1 | 70 | 38.16 | 1.04 | 99 | 47.33 | 2.08 |
| 4 | 75 | 66.66 | 1.75 | 103 | 31.33 | 1.52 |
| 5 | 80 | 71.33 | 1.52 | 110 | 20.66 | 1.52 |
| 6 | 85 | 88.33 | 2.08 | 115 | 10 | 1 |
| 7 | 90 | 88.66 | 1.52 | 120 | 8 | 1 |

**Table S2. (**Continued)

| **SPHHs-AT** | Time ^min^ | pH-1.2 (AV) | pH-1.2 (SD) | Time ^min^ | pH-7.4 (AV) | pH-7.4 (SD) |
| --- | --- | --- | --- | --- | --- | --- |
| 3 | 120 | 7.5 | 0.5 | 150 | 87.66 | 1.52 |
| 2 | 125 | 26 | 1 | 155 | 66.66 | 1.52 |
| 1 | 130 | 39 | 2 | 160 | 56.33 | 1.52 |
| 4 | 135 | 63 | 1 | 165 | 27.16 | 1.75 |
| 5 | 140 | 76 | 1 | 170 | 22 | 1 |
| 6 | 145 | 79.5 | 1.3 | 175 | 11.83 | 1.6 |
| 7 | 150 | 87.5 | 1.5 | 180 | 5.5 | 1.32 |

**Table S2. (**Continued)

| SPHHs-AT | **Time ^min^** | **pH-1.2 (AV)** | **pH-1.2 (SD)** | **Time ^min^** | **pH-7.4 (AV)** | **pH-7.4 (SD)** |
| --- | --- | --- | --- | --- | --- | --- |
| **3** | 180 | 8.33 | 1.52 | 210 | 87.33 | 1.52 |
| **2** | 185 | 27.5 | 1.32 | 215 | 71.83 | 1.6 |
| **1** | 190 | 41.5 | 1.5 | 220 | 42 | 2 |
| **4** | 195 | 72.5 | 1.5 | 225 | 25.16 | 1.04 |
| **5** | 199 | 76.5 | 1.32 | 230 | 21 | 1 |
| **6** | 205 | 80.33 | 1.52 | 235 | 14 | 1 |
| **7** | 210 | 87.33 | 2.01 | 240 | 8.33 | 2.01 |

Herein,.
SPHHs-AT-3 by the interchange of the swelling tool between (pH 1.2) HCl solutions and (pH 7.4) phosphate
buffer solutions (n =3, mean ± S.D); AV/SD= Average and standard deviation.

**Table S3.** Experimental data for gastroretentive SPHHs formulations.

| **Sample (SPHHs)** | **Density (g/cm3)^†^** | **Sol–gel time (s)** | **Compressive strength (N/m2) (n =3)** | **% Drug content (n =3) SPHHs-AT (mean ± SD)** |
| --- | --- | --- | --- | --- |
| **1** | 0.651 ± 0.015 | 24 ± 1 | 5756.093 ± 29.783 | 95.646 ± 0.674 |
| **2** | 0.568 ± 0.015 | 25 ± 1 | 4310.167 ± 17.737 | 96.943 ± 1.008 |
| **3** | 0.503 ± 0.036 | 26 ± 1 | 4074.477 ± 20.636 | 98.053 ± 0.319 |
| **4** | 0.684 ± 0.04 | 31 ± 1 | 6231.867 ± 29.613 | 97.093 ± 0.305 |
| **5** | 0.747 ± 0.03 | 27 ± 1 | 7259.207 ± 50.028 | 96.383 ± 0.380 |
| **6** | 0.798 ± 0.026 | 30 ± 1 | 7881.501 ± 40.606 | 95.363 ± 0.536 |
| **7** | 0.844 ± 0.03 | 32 ± 1 | 8273.797 ± 30.495 | 96.393 ± 0.183 |

† The forceps concept was used for density measurement, and a predetermined hexane volume was submerged for polymer [13].

References

1. Balamuralidhara, V., Pramod Kumar, T., Vishal Gupta, N., Getyala, A. & Gangadharappa, H. Development of a novel biodegradable superporous hydrogel for gastroretentive application. International Journal of Polymeric Materials and Polymeric Biomaterials **62**, (2013), 524-532 . https://doi.org/10.1080/00914037.2012.735297

2. Bashir, S. et al. Fundamental concepts of hydrogels: Synthesis, properties, and their applications. Polymers **12**, (2020), 2702 . https://doi.org/10.3390/polym12112702

3. Hibbins, A. R. et al. Design of a versatile pH-responsive hydrogel for potential oral delivery of gastric-sensitive bioactives. Polymers **9**, (2017), 474. https://doi.org/10.3390/polym9100474

4. Khan, F. et al. Synthesis, classification and properties of hydrogels: their applications in drug delivery and agriculture. Journal of Materials Chemistry B **10**, (2022), 170-203. https://doi.org/10.1039/D1TB01345A

5. Omidian, H. & Park, K. Superporous hydrogels for drug delivery systems, 2017, Faculty Books and Book Chapters. 15,2, Elsevier, 978-0-08-100692-4, 688-704.

6. Qiu, Y. & Park, K. Superporous IPN hydrogels having enhanced mechanical properties. AAPS PharmSciTech **4**, (2003), 406-412. https://doi.org/10.1208/pt040451

7. Zhang, H. et al. Bio-Inspired Preparation of Clay–Hexacyanoferrate Composite Hydrogels as Super Adsorbents for Cs+. ACS applied materials & interfaces **12**, (2020), 33173-33185. https://doi.org/10.1021/acsami.0c06598

8. Farid-ul-Haq, M. et al. A smart drug delivery system based on Artemisia vulgaris hydrogel: Design, on-off switching, and real-time swelling, transit detection, and mechanistic studies. Journal of Drug Delivery Science and Technology **58**, (2020), 101795. https://doi.org/10.1016/j.jddst.2020.101795

9. Hussain, M. A., Rana, A. I., Haseeb, M. T., Muhammad, G. & Kiran, L. Citric acid cross-linked glucuronoxylans: A pH-sensitive polysaccharide material for responsive swelling-deswelling vs various biomimetic stimuli and zero-order drug release. Journal of Drug Delivery Science and Technology **55**, (2020), 101470. https://doi.org/10.1016/j.jddst.2019.101470

10. Raza, M. A., Lim, Y.-M., Lee, S.-W., Seralathan, K.-K. & Park, S. H. Synthesis and characterization of hydrogels based on carboxymethyl chitosan and poly (vinylpyrrolidone) blends prepared by electron beam irradiation having anticancer efficacy, and applications as drug carrier for controlled release of drug. Carbohydrate Polymers **258**, (2021), 117718. https://doi.org/10.1016/j.carbpol.2021.117718

11. Hao, N., Jayawardana, K. W., Chen, X. & Yan, M. One-step synthesis of amine-functionalized hollow mesoporous silica nanoparticles as efficient antibacterial and anticancer materials. ACS applied materials & interfaces **7**, (2015), 1040-1045, https://doi.org/10.1021/am508219g

12. Bardonnet, P., Faivre, V., Pugh, W., Piffaretti, J. & Falson, F. Gastroretentive dosage forms: Overview and special case of Helicobacter pylori. Journal of controlled release **111**, (2006), 1-18. https://doi.org/10.1016/j.jconrel.2005.10.031

13. Gümüşderelioğlu, M., Erce, D. & Demirtaş, T. T. Superporous polyacrylate/chitosan IPN hydrogels for protein delivery. Journal of Materials Science: Materials in Medicine **22**, (2011), 2467-2475. https://doi.org/10.1007/s10856-011-4422-4
